# Building Research and Development Capacity for Neglected Tropical Diseases Impacting Leishmaniasis in the Middle East and North Africa: A Case Study

**DOI:** 10.1371/journal.pntd.0003695

**Published:** 2015-08-27

**Authors:** Sima Rafati, Shaden Kamhawi, Jesus G. Valenzuela, Mostafa Ghanei

**Affiliations:** 1 Department of Immunotherapy and *Leishmania* Vaccine Research, Pasteur Institute of Iran, Tehran, Iran; 2 Vector Molecular Biology Section, Laboratory of Malaria and Vector Research, National Institute of Allergy and Infectious Diseases, National Institutes of Health, Rockville, Maryland, United States of America; 3 Director Office, Pasteur Institute of Iran, Tehran, Iran; University of Notre Dame, UNITED STATES

There is an urgent need to build Research and Development (R&D) capacity for leishmaniasis and other neglected tropical diseases (NTDs) in the Middle East and North Africa.

The neglected tropical diseases are a group of 17 viral, bacterial, protozoan, and helminthic infections that often have high morbidity but low mortality, causing disabilities through their impact on child development and education, pregnancy outcomes, and worker productivity [1].

NTDs promote poverty and interfere with economic development. Emerging information indicates that the Middle East and North Africa (MENA) region is highly endemic for several NTDs, with increasing levels of poverty and disease in the region [2]. Today, with the exception of Buruli ulcer, Chagas disease, dracunculiasis, and human African trypanosomiasis, the 13 remaining NTDs are found in MENA countries, with the most prevalent (both on and off the WHO’s list) being dengue virus, rabies, brucellosis, leprosy, trachoma, toxoplasmosis, cutaneous and visceral leishmaniasis, toxoplasmosis, facioliasis, schistosomiasis, and soil-transmitted helminth infections [2,3]. Nations such as Algeria, Iran, Libya, Morocco, Syria, Egypt, and Yemen are impoverished countries with especially high rates of NTDs [4]. Among all, *Leishmania* is the most important protozoan infection in the MENA region [5]. Rapid urbanization and human migration in this region has led to the spread of the sand fly vector and, consequently, leishmaniasis. Despite establishing national programs for sand fly vector control and leishmaniasis treatment, the disease continues to spread. Almost two-thirds of cutaneous leishmaniasis (CL) cases are reported from Afghanistan, Algeria, Brazil, Colombia, Iran, Syria, and Iraq [6]. Among the different countries of the MENA region, Syria is known to have the highest prevalence of CL (nearly 52,983 cases were reported in 2012). Incidence of visceral leishmaniasis (VL) in MENA is sporadic and has been reported mostly from Iraq (nearly 1,041 cases were reported in 2008), where 90% of cases occur in children under five years of age [6].

Globally, compared to HIV/AIDS, malaria, and tuberculosis, which receive 42.1% of the funds allocated to overseas development assistance for health, NTDs have generally been ignored, receiving only 0.6% of the budget [7]. Studies have also established that financial support for Research and Development for NTDs significantly lags behind AIDS and other better-known diseases [8]. While global efforts to eliminate some NTDs, such as lymphatic filariasis (http://www.afro.who.int/), have been successful in MENA countries, many have not received such attention.

A similar level of neglect for both mass treatment of and R&D for NTDs has been noted for MENA countries. The World Health Organization (WHO) reports that mass drug administration coverage for many NTDs in MENA is low. For instance, the coverage for soil-transmitted helminthic infections in the WHO Eastern Mediterranean Region is less than one-half the coverage in sub-Saharan Africa [9]. In addition to mass treatments, we need to improve diagnosis and control of NTDs in MENA countries. This can be achieved by promoting R&D of new tools, including diagnostics and vaccines, and addressing challenges facing their implementation. So, how can we share critical information and identify the major obstacles to translating scientific breakthroughs into innovative strategies for reducing the burden of NTDs in MENA countries? We can achieve this goal by building research capacity that will promote support for the translation of laboratory findings into field applications, a crucial component for controlling the NTDs in the MENA region. Additionally, intervention and implementation research should focus on resolving barriers particular to MENA countries and translate into proper approaches for effective delivery of health interventions aimed at control or elimination of the targeted diseases. For example, a rapid response to outbreaks is generally poor in endemic MENA countries due to the absence of a good infrastructure for surveillance as well as lack of funding. In some cases, this is compounded by conflict and mass migrations that compromise the control of disease outbreaks in their early stages. In summary, two major tasks need to be undertaken to improve the control of NTDs in MENA: (i) increase research capacity in endemic MENA countries and (ii) translate laboratory discoveries into field applications appropriate for endemic MENA countries.

As mentioned previously, leishmaniasis represents a good case study of the challenges facing control of NTDs in MENA. Today, MENA countries exhibit some of the highest incidences of cutaneous leishmaniasis globally [4], with worrisome increases in the war-torn areas of Syria, Iraq, and elsewhere. Besides the long-standing and crucial support of WHO-TDR (the Special Programme for Research and Training in Tropical Diseases), aimed at fostering an effective global research effort to control infectious diseases and promoting the translation of innovation to impact health in the MENA region, there are few other scientific activities that promoted leishmaniasis R&D in MENA countries during the past few years. A research and policy conference, LEISHMANIA: Collaborative Research Opportunities in North Africa and the Middle East, was held in June 2009 in Tunisia to promote international collaboration between the United States and the MENA countries most affected by leishmaniasis [10]. The conference was supported by the US National Institute of Allergy and Infectious Diseases Office of Global Research (OGR) and the National Institutes of Health (NIH) and hosted locally by the Institute Pasteur de Tunis. The developed collaborations and the outcome of this conference resulted in several successful grant proposals funded by the Civilian Research and Development Foundation from the OGR [11,12]. This remains only one successful example focused on leishmaniasis and there is a need for more of such supportive conferences to promote NTD research, especially in the MENA region. In July 2014, a European and Developing Countries Clinical Trials Partnership (EDCTP) forum on capacity development in Africa was held in Berlin. EDCTP was created in 2003 as a European response to the global health crises caused by three poverty-related diseases, including HIV/AIDS, tuberculosis, and malaria. The objective of the meeting was to identify current and emerging capacity development gaps in order to inform the development of the strategy and operational plans to control poverty-related diseases [13]. Similar to EDCTP, identifying the capacity development gaps in the MENA region, especially for highly endemic diseases such as cutaneous leishmaniasis, is highly recommended. Furthermore, creating a harmonized regional network to share information and experiences on different aspects of leishmaniasis is essential. These types of activities will help and promote opportunities to advise health authorities about the most effective measures for prevention and control of leishmaniasis, one of the highly prevalent diseases in MENA countries.

In addition to conferences, by establishing regional R&D and training centers of excellence in the MENA region, we could promote and build long-lasting research skills and capacities and enhance the contribution of research to the control and elimination of NTDs. One such center was suggested recently for southern Europe [14], but a parallel center needs to be created in the MENA region. Such a training center could act as a resource for regional training activities that provide the building blocks for doing solid research on NTDs. It could also improve the ability of young researchers to establish a global network that promotes collaboration between institutions of the MENA region. By establishing a regional training center, sustainable capacity could be built through exchanging experiences and innovative approaches. Such a center would also foster good practices in health research to be commonly implemented by the researchers in this region.

Iran is one of the MENA countries with the highest prevalence of zoonotic cutaneous leishmaniasis (*Leishmania major*), and the second-highest prevalence of anthroponotic cutaneous leishmaniasis (*Leishmania tropica* infection) [4]. With more than a decade of support from WHO-TDR, Iran led the development of first-generation leishmanization activities, which provide the underpinnings for more advanced vaccine development [15]. There are different universities and institutes in Iran with excellent scientific infrastructures that could act as a regional training center for leishmaniasis and other NTDs in the MENA region. Among all, Pasteur Institute of Iran is the oldest; it was established in 1920 in Tehran, using the Pasteur Institute of Paris as a template. Pasteur Institute of Iran has a close and active cooperation with international organizations such as the WHO, WHO-TDR, United Nations Children’s Fund (UNICEF), NIH, Drugs for Neglected Diseases initiative (DNDi), and the Paris Institut Pasteur. The Pasteur Institute of Iran is a member of the Pasteur International Network and has well-established links to Pasteur institutions in Paris, Tunisia, and Morocco. Increased investment in these types of research institutes will greatly advance research capacity building in the MENA region. Strengthening the connections between education, research, and the international scientific community can have a tremendous, positive effect on this region. By creating an active scientific atmosphere (for instance, via research training centers), established researchers will be motivated and young graduates will be attracted to careers in scientific research, improving the overall capability of the MENA region to control NTDs.

In the coming years, geopolitical events taking place in the MENA region could promote the emergence or re-emergence of widespread NTDs. Now, more than ever, we need to re-examine the necessity to strengthen and expand existing programs for the control of NTDs in MENA, through improvement of mass treatment coverage for these diseases and identification of innovative ways to enhance R&D capacity in the region.
